# Assessment of growth and metabolism characteristics in offspring of dehydroepiandrosterone-induced polycystic ovary syndrome adults

**DOI:** 10.1530/REP-16-0081

**Published:** 2016-10-20

**Authors:** Ying Huang, Jiang-Man Gao, Chun-Mei Zhang, Hong-Cui Zhao, Yue Zhao, Rong Li, Yang Yu, Jie Qiao

**Affiliations:** 1 Reproductive Medical CenterDepartment of Obstetrics and Gynecology, Peking University Third Hospital, Beijing, China; 2 Key Laboratory of Assisted ReproductionMinistry of Education, Beijing, China; 3 Beijing Key Laboratory of Reproductive Endocrinology and Assisted Reproductive TechnologyBeijing, China

## Abstract

Polycystic ovary syndrome (PCOS) is a common reproductive disorder that has many characteristic features including hyperandrogenemia, insulin resistance and obesity, which may have significant implications for pregnancy outcomes and long-term health of women. Daughters born to PCOS mothers constitute a high-risk group for metabolic and reproductive derangements, but no report has described potential growth and metabolic risk factors for such female offspring. Hence, we used a mouse model of dehydroepiandrosterone (DHEA)-induced PCOS to study the mechanisms underlying the pathology of PCOS by investigating the growth, developmental characteristics, metabolic indexes and expression profiles of key genes of offspring born to the models. We found that the average litter size was significantly smaller in the DHEA group, and female offspring had sustained higher body weight, increased body fat and triglyceride content in serum and liver; they also exhibited decreased energy expenditure, oxygen consumption and impaired glucose tolerance. Genes related to glucolipid metabolism such as *Pparγ*, *Acot1/2*, *Fgf21*, *Pdk4* and *Inhbb* were upregulated in the liver of the offspring in DHEA group compared with those in controls, whereas *Cyp17a1* expression was significantly decreased. However, the expression of these genes was not detected in male offspring. Our results show that female offspring in DHEA group exhibit perturbed growth and glucolipid metabolism that were not observed in male offspring.

## Introduction

Polycystic ovary syndrome (PCOS) is a common reproductive disorder with many characteristics, including hyperandrogenemia, insulin resistance and obesity, which may have significant implications for pregnancy outcomes and long-term health of women. Familial history of PCOS represents a risk factor for development of the disorder, as first-degree relatives of patients with PCOS have higher prevalence of PCOS (Legro *et al.* 1998, Kahsar-Miller *et al.* 2001). Familial aggregation of PCOS kindreds suggests the involvement of a major genetic component(s) in this disorder. However, the results of subsequent genetic studies have been inconsistent and cannot fully explain the pathogenesis of PCOS.

PCOS women have increased risk of prenatal and neonatal complications such as gestational diabetes, pregnancy-induced hypertension, pre-eclampsia and preterm birth (Boomsma *et al.* 2006). Also, infants of women with PCOS are more likely to be large or small for gestational age (Sir-Petermann *et al.* 2005, Anderson *et al.* 2010). In a two-period study, female infants and prepubertal girls born to PCOS mothers were found to have significantly higher levels of anti-Mullerian hormone (AMH), whereas the concentrations of gonadotropin and sex steroids in both PCOS groups were comparable with those of control groups (Sir-Petermann *et al.* 2006). The observed higher levels of AMH in PCOS daughters were found to continue into adolescence (Crisosto *et al.* 2007). AMH is produced by granulosa cells and reflects follicular development. As such, the increase in AMH suggests that daughters of PCOS women may have altered follicular development during infancy and childhood (Crisosto *et al.* 2007).

Prepubertal and pubertal daughters of PCOS women have a normal body mass index and Tanner stage distribution but have some hormonal perturbations and differences in metabolic parameters (Sir-Petermann *et al.* 2007). Hyperinsulinemia and an increased ovarian volume occur with PCOS before the onset of puberty and persist during pubertal development (Sir-Petermann *et al.* 2009). Considering the early onset and the nature of the metabolic and sex hormone alterations, PCOS daughters constitute a high-risk group for metabolic and reproductive derangements (Sir-Petermann *et al.* 2007, 2009). Owing to the complexity of the multiple factors that induce PCOS, a comprehensive evaluation of PCOS offspring has not been reported. Studies of PCOS offspring have been conducted only with small sample sizes and included a single ethnic group (Crisosto *et al.* 2007, Sir-Petermann *et al.* 2007). The follow-up period only reached adolescence—a period of rapid change and hormonal fluctuations—and thus is not representative of hormone metabolism or cannot completely represent the hormone metabolism situation in adulthood.

Androgen excess is the most common defect underlying PCOS, and it manifests in 25–60% of patients. PCOS patients have elevated levels of adrenal androgens, particularly dehydroepiandrosterone (DHEA), its sulfoconjugate and androstenedione (Carmina *et al.* 1992, Moran *et al.* 1999). Phenotypic and metabolic variations among PCOS patients lead to substantial variation in results.

In this study, we investigated the growth and metabolic indices of offspring conceived from DHEA-induced PCOS mice to illuminate their developmental characteristics and determine the expression profiles of key genes. Our results provide a fundamental basis for comprehending potential health issues affecting PCOS offspring.

## Materials and methods

All chemicals used in this study were purchased from Sigma-Aldrich Chemical Company unless otherwise stated.

### Ethics approval

All procedures involving mice were carried out following strict criteria of the Guide for Care and Use of Laboratory Animals of Peking University, and the protocol was approved by the Institutional Animal Care and Use Committee of Peking University Third Hospital.

### PCOS-like model

Female prepubertal (25-days old) mice of the BALB/c strain (Vital River Laboratories, Beijing, China) were randomly divided into the DHEA group and control group. The PCOS-like mouse model was established by subcutaneously injecting DHEA (6 mg/100 g body weight dissolved in 0.05 mL sesame oil) once daily for 20 consecutive days as described (Huang *et al.* 2015). All mice were raised and housed in the Animal Center of the Medical College of Peking University according to the national legislation for animal care. All mice were maintained under controlled temperature and lighting conditions and allowed free access to food and water. After 20 days of treatment, eight to ten mice per group were killed for model validation (Huang *et al.* 2015).

### Offspring acquisition

Mice from both control and DHEA groups were superovulated via intraperitoneal injection of 10 IU of equine chronic gonadotropin (Hua Fu Biotechnology, Tianjin, China), followed by 10 IU of human chorionic gonadotropin (HCG, Hua Fu Biotechnology) 48 h later. On the same day, equine chronic gonadotropin was given, DHEA administration was stopped. DHEA and control mice were paired with BALB/c males (6–8 weeks of age) in the evening of HCG injection, and the females with confirmed vaginal plugs the next morning were considered pregnant. Spontaneous delivery was allowed, and offspring were weaned at age 3 weeks. Female and male offspring were fed separately until age 12 weeks and weighed weekly. All offspring were maintained under controlled temperature and lighting conditions and allowed free access to food and water. Metabolic status and reproductive function were assessed at age 12–13 weeks, with the exception of estrous cycle evaluation, which was performed at age 6 weeks.

### Body composition analysis

To determine body fat mass composition, offspring were placed in a clear plastic holder without anesthesia or sedation and inserted into an EchoMRI device (Echo Medical Systems, Houston, TX, USA) at age 12–13 weeks. Eight female and eight male mice were assessed per group.

### Indirect calorimetry

Energy expenditure was measured using an indirect open-circuit calorimeter (Oxylet; Panlab, Spain). Mice were placed individually into metabolic cages for 48 h. After a 24-h acclimation period, oxygen consumption (VO_2_) and carbon dioxide production (VCO_2_) were monitored for 3 min every hour in each cage for 24 h. At the same time, locomotor activity was monitored by Activity Sensor (Oxylet), and these data were collected every 2 min. Energy expenditure (in kcal/day/kg^0.75 ^= [3.815 + 1.232 × RQ] × VO_2_ × 1.44) and respiratory quotient (RQ = VCO_2_/VO_2_) were calculated using METABOLISM software (Oxylet). Eight female and eight male mice per group were assessed at age 12–13 weeks.

### Blood pressure measurement

The blood pressure of offspring (eight mice per group) was measured with a CODA noninvasive blood pressure meter (Kent Scientific, Torrington, CT, USA) at age 12–13 weeks.

### Oral glucose tolerance test

An oral glucose tolerance test was performed in mice following an 8 h fast. Glucose levels were measured in tail-vein blood samples using a blood glucose meter (Sinocare, Changsha, China). After the fast, glucose levels were measured, and then glucose (2 g/kg body weight dissolved in 10 mL water) was administered to mice by oral gavage, and tail-vein samples were obtained at 30, 60, 90 and 120 min after administration.

### Blood sampling for insulin, leptin and lipid profiles and measurement of hepatic cholesterol and triglycerides

At age 12–13 weeks, eight female and male offspring in each group were killed. Blood was drawn from the inner canthus after the mice were fasted for 8 h. The serum was immediately separated and stored at −80°C for subsequent analysis or hormone determinations. Leptin and fasting insulin levels were measured with radioimmunoassay kits (Beijing North Institute of Biological Technology, Beijing, China). Lipid profiles were determined using biochemical analysis kits (China Diagnostics Medical Corporation, Beijing, China). After blood sample collection, each liver was immediately removed and stored at −80°C. Total cholesterol (CHO) and triglycerides (TG) were extracted from liver and measured with a Tissue Total Cholesterol Assay Kit and Tissue Triglyceride Assay Kit (Applygen Technologies, Beijing, China).

### Quantitative real-time PCR

Quantitative real-time PCR was used to determine the levels of the expression of glucolipid metabolism–related genes in liver. Total RNA from the liver was isolated with TRIzol reagent (Invitrogen). cDNA was synthesized using the First Strand cDNA Synthesis kit (Thermo Fisher). For quantitative PCR, amplification was performed with an ABI7500 (Applied Biosystems) using the SYBR Green kit (Applied Biosystems). The cycling conditions were 2 min at 95°C, followed by 40 cycles of 30 s at 95°C and 1 min at 60°C for annealing. GAPDH served as the internal control for gene expression normalization. The relative expression levels of the glucolipid metabolism–related genes were calculated using the 2^−ΔΔCT^ method. All primers were synthesized by Sangon Company, and the sequences are shown in Supplementary Table 1, see section on supplementary data given at the end of this article.

### Statistical analysis

Measurement data were presented as the mean ± s.e.m. Statistical significance was determined as indicated by two independent sample *t*-tests, Mann–Whitney *U* test and *χ*^2^ tests, using SPSS 16.0 software appropriately. A *P* value of <0.05 was considered to reflect a statistically significant difference.

## Results

### Producing offspring by superovulating DHEA-induced PCOS female mice

DHEA-induced PCOS female mice and control female mice were mated with normal BALB/c male mice (a single male mouse per female). The pregnancy rate in the DHEA group was only 8% (compared with 67% for the control group), which was too low to obtain sufficient offspring; hence, in subsequent experiments, hormone therapy was used to stimulate ovulation.

In the control group, a vaginal plug was observed in 10 mice after stimulating ovulation and mating, and 7 mice produced offspring (70%); average litter size was 7.6 ± 1.0. In the DHEA group, a vaginal plug was observed in 13 mice, and 6 mice produced offspring (46%); average litter size was 4.2 ± 2.3, which was significantly smaller than that of the control group (*P <* 0.05).

### Growth index comparison between offspring of DHEA and control mice

**Figure 1:**
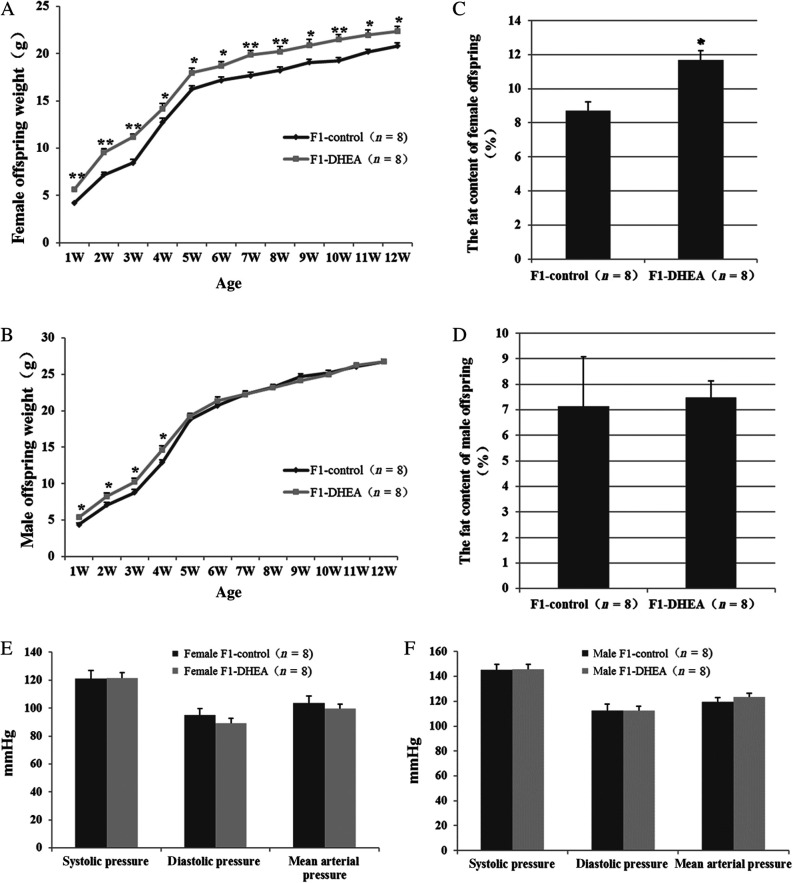
Growth of offspring of DHEA and control mice. (A and B) Growth curves for female offspring (A) and male offspring (B) during postnatal weeks 1–12. (C and D) Fat content of female offspring (C) and male offspring (D) at age 12 weeks. (E and F) Systolic, diastolic and mean arterial pressure of female (E) and male (F) offspring. Values are the mean ± s.e.m., and n denotes the number of mice. *P < 0.05, **P < 0.01 vs control group.

As shown in Fig. 1A, the average body weight of female offspring of DHEA mice was significantly greater than that of the control group from one week after birth until adulthood (12 weeks). During postnatal weeks 1–4, the body weight of male offspring in the DHEA group also was significantly greater than that of male offspring in the control group. However, no difference in male offspring body weight was observed between the two groups from postnatal weeks 5–12 (Fig. 1B).

Fat mass of the offspring at age 12 weeks was measured by magnetic resonance imaging. The mean fat mass percentage for female offspring was 11.69 ± 0.55% in the DHEA group and 8.71 ± 0.52% in the control group, a difference that was statistically significant (Fig. 1C). The mean fat mass of male offspring did not differ significantly between the two groups (7.48 ± 0.66% vs 7.15 ± 1.92% respectively, Fig. 1D).

As metabolic disorders often leads to vascular endothelial dysfunction and increased risk of hypertension, blood pressure was measured in adult offspring. For both female and male offspring, there were no significant differences in systolic, diastolic or mean arterial pressure between the DHEA and control groups (Fig. 1E and F).

### Comparison of metabolic level between offspring of DHEA and control mice

Obesity is often caused by excessive food intake or metabolic changes, resulting in weight gain and pathological changes induced by excess body fat accumulation. Energy consumption and activity levels can indirectly reflect the metabolic levels and provide an explanation for weight change. In this study, dynamic changes in energy consumption (energy expenditure), VO_2_, VCO_2_ and activity were monitored with a respiratory metabolism detector for 24 h for eight female and eight male offspring from each group.

**Figure 2:**
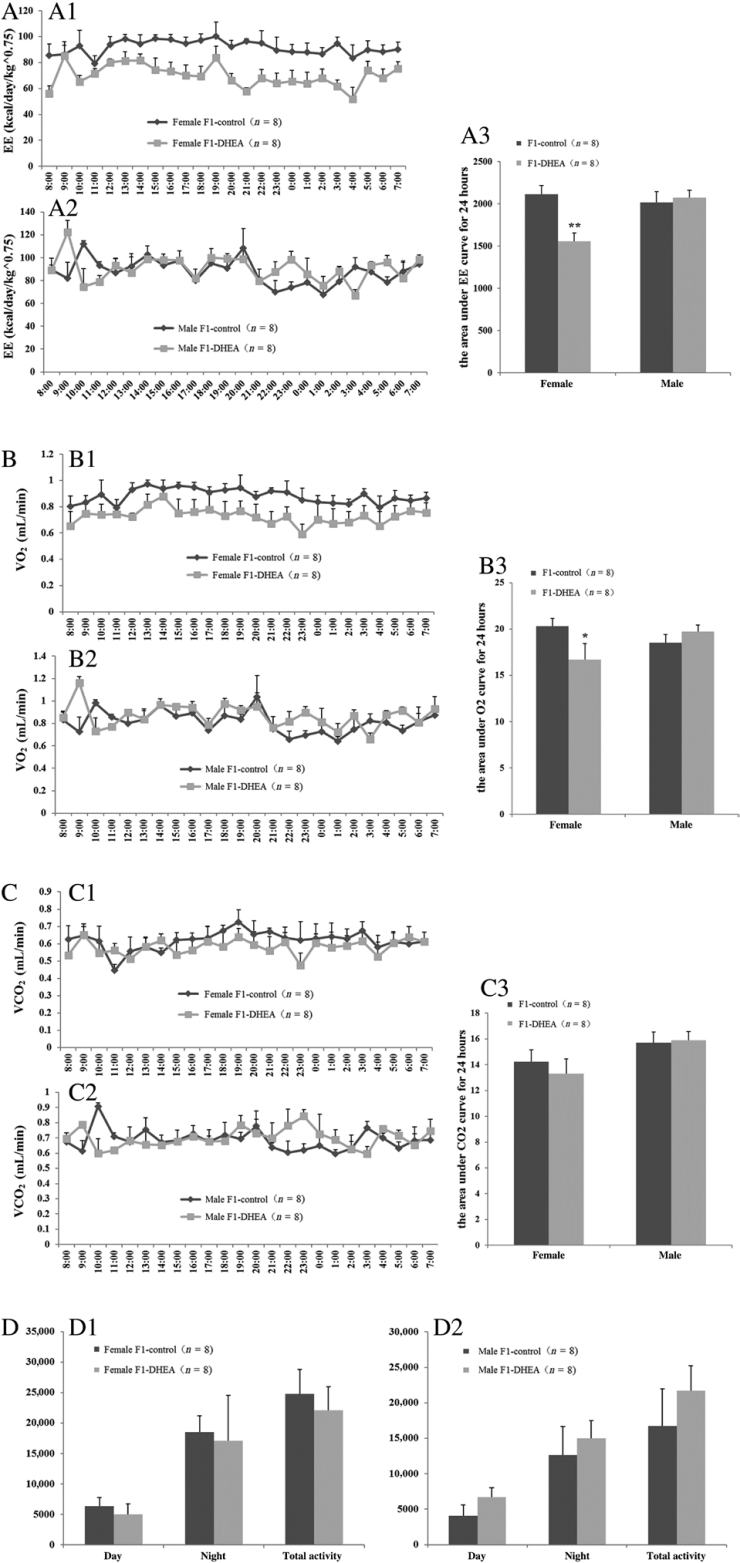
Metabolism level in offspring of DHEA and control mice. (A) Energy consumption: A1, female offspring, dynamic change of energy consumption in 24-h; A2, male offspring, dynamic change of energy consumption in 24-h; A3, area under the curve (AUC) for energy consumption in 24 hours in female and male offspring. (B) Oxygen consumption: B1, female offspring, dynamic change of O_2_ consump­tion in 24-h; B2, male offspring, dynamic change of O_2_ consumption in 24-h; B3, AUC for O_2_ consumption in 24 h in female and male offspring. (C) CO_2_ production: C1, female offspring, dynamic change of CO_2_ production in 24 h; C2, male offspring, dynamic change of CO_2_ production in 24 h. C3, AUC for CO_2_ production in 24 h in female and male offspring; (D) Activity: D1, female offspring, activity during the day, night and over a 24-h period; D2, male offspring activity during the day, night and over a 24-h period. ‘*n*’ denotes the number of mice. *P < 0.05, **P  < 0.01 vs control group.

Data for energy expenditure were adjusted for body weight. Female offspring of DHEA mothers showed sustained lower levels of energy consumption and VO_2_ (Fig. 2A and B). No difference was observed for male offspring. Moreover, VCO_2_ and physical activity did not differ significantly in female/male offspring of DHEA and control mice at any time over the 24-h period (Fig. 2C and D).

**Figure 3:**
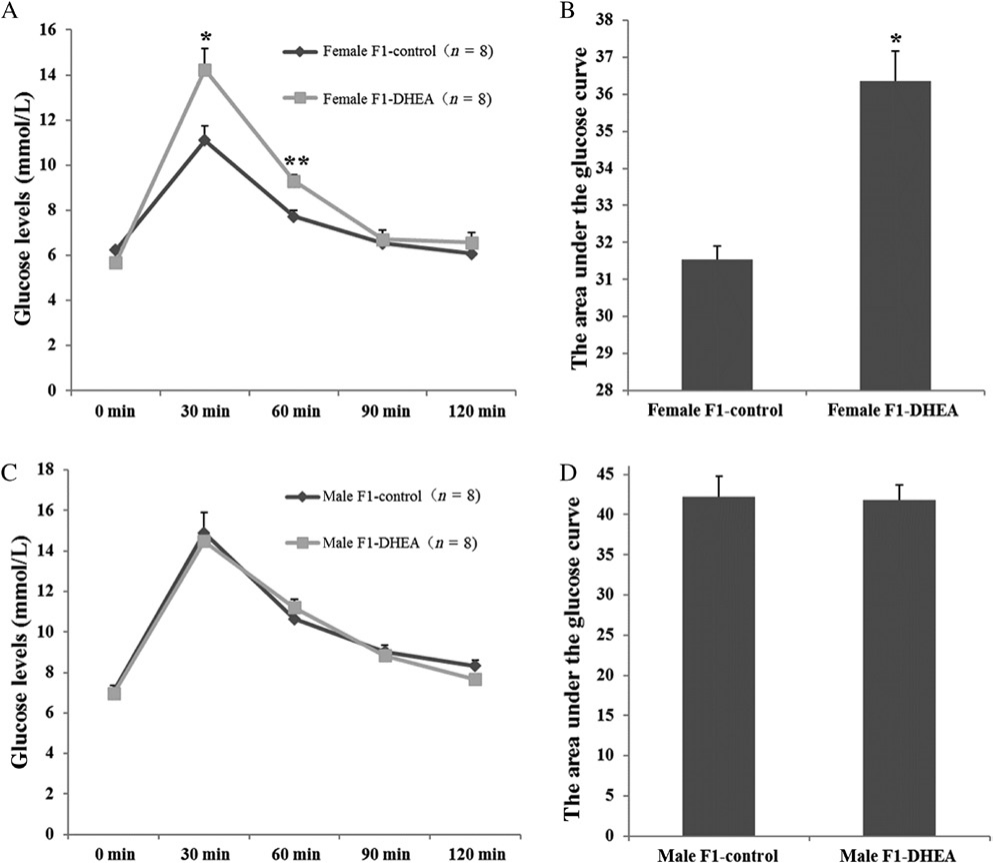
Oral glucose tolerance test (OGTT) for offspring of DHEA and control mice. (A) Blood glucose level curves for female offspring at each time point. (B) OGTT area under the curve for female offspring. (C) Blood glucose level curves for male offspring at each time point. (D) OGTT area under the curve for male offspring. ‘*n*’ denotes the number of mice. *P < 0.05, **P < 0.01 vs control group.

An oral glucose tolerance test was performed to assess insulin sensitivity in adult offspring. In female offspring, the glucose level at both 30 and 60 min after the sugar–water lavage was significantly higher in the DHEA group than that in the control group. Moreover, the area under the curve for glucose was also significantly greater, suggesting impaired glucose tolerance in DHEA female offspring. However, compared with control males, the glucose tolerance of the male offspring was unaffected, with comparable blood glucose levels at each time point (Fig. 3).

Fasting serum lipids and metabolism–related hormone levels in offspring were measured in 12-week-old mice. Except for serum TG level, which was significantly higher in the DHEA female offspring, other indicators, such as HOMA-IR, cholesterol, HDL, LDL, insulin and leptin levels, did not differ significantly between the two groups (Table 1).

**Table 1:** Lipid and insulin level in offspring of DHEA and control mice.

	**Female F1-control** (*n *= 8)	**Female F1-DHEA** (*n *= 8)	**Male F1-control (*n *= 8)**	**Male F1-DHEA (*n *= 8)**
Total CHO (mmol/L)	1.63 ± 0.12	1.53 ± 0.14	2.40 ± 0.13	2.19 ± 0.19
TG (mmol/L)	0.95 ± 0.05	2.21 ± 0.35*	1.08 ± 0.12	1.34 ± 0.13
HDL (mmol/L)	1.29 ± 0.10	1.26 ± 0.12	1.82 ± 0.08	1.75 ± 0.16
LDL (mmol/L)	0.19 ± 0.01	0.18 ± 0.01	0.23 ± 0.02	0.22 ± 0.01
FBG (mmol/L)	5.10 ± 0.41	4.40 ± 0.20	6.52 ± 0.39	6.58 ± 0.22
FINS (μIU/mL)	10.31 ± 0.65	11.07 ± 0.79	14.34 ± 0.85	14.58 ± 1.20
HOMA-IR	2.55 ± 0.23	2.18 ± 0.22	4.16 ± 0.37	4.30 ± 0.44
Leptin (ng/mL)	7.94 ± 0.20	7.06 ± 0.88	5.55 ± 0.69	5.04 ± 0.27

* *P *< 0.05, vs control group.

**Figure 4:**
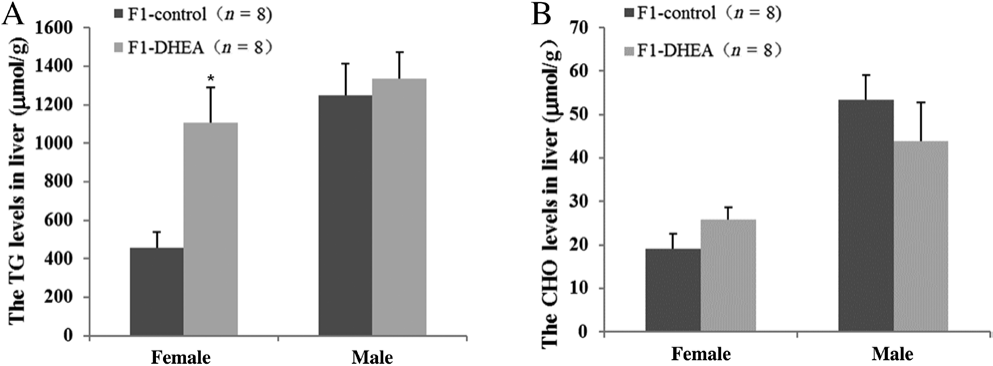
TG and CHO content of liver and muscle tissue in offspring of DHEA and control mice. (A) TG content in liver tissue. (B) Total CHO content in liver tissue. ‘*n*’ denotes the number of mice. *P < 0.05 vs control group.

The liver is the central hub of human metabolism, regulating both glucose and lipid metabolism. We thus measured CHO and TG levels in the liver. Mean liver TG concentration was 1108.7 ± 183.4 μmol/g in the DHEA female offspring, which was significantly greater than that of female controls (455.8 ± 82.32 μmol/g); total CHO, however, was similar in the two groups (Fig. 4A and B). For the male offspring, there was no difference in liver TG or CHO levels between the two groups (Fig. 4A and B).

### Expression of genes that regulate glucolipid metabolism in liver tissue

**Figure 5:**
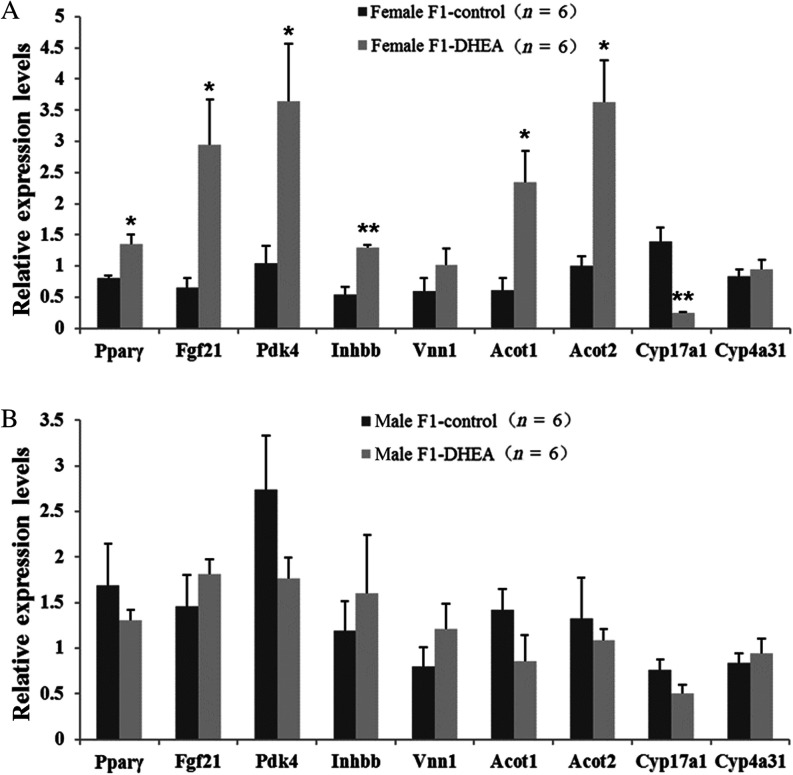
Real-time PCR assay of differential expression genes in liver of offspring. (A) Female offspring. (B) Male offspring. ‘*n*’ denotes the number of mice. *P < 0.05, **P < 0.01 vs control group.

To determine the potential molecular mechanism underlying the observed dynamic metabolic changes in PCOS female mice, we used real-time PCR to assess the expression of 36 glucolipid metabolism–related genes in liver tissue of female offspring of DHEA and control mothers (three cases per group). Among these genes, 17 were associated with lipid synthesis, transport and metabolism (*Acc*, *Acs*, *CD36*, *Cpt-2*, *Cyp17a1*, *Fas*, *Fatp*, *Fgf21*, *Hmgcl*, *Hmgcs2*, *Inhbb*, *Lepr*, *Pparα*, *Pparβ*, *Pparγ*, *Scd* and *Vnn1*; Smith 2002, Lee *et al.* 2003, Yoon 2009, Rakhshandehroo *et al.* 2010, Nakamura *et al.* 2014), 6 were associated with glucose synthesis and metabolism (*Aqp3*, *Glut4*, *Gyk*, *Pck1*, *Pdk4* and *Pepck*; Lee *et al.* 2003, Yoon 2009, Nakamura *et al.* 2014) and 13 were associated with oxidation and decomposition (*Acaa1a*, *Acot1*, *Acot2*, *Acox1*, *Aldh3a1*, *Cpt1A*, *Cyp4a1*, *Cyp4a31*, *Etfdh*, *Hadha*, *Hsd17b4*, *Ucp2* and *Ucp3*; Lee *et al.* 2003, Yoon 2009, Nakamura *et al.* 2014). Preliminary screening results showed that, for the female offspring, 9 genes (*Acot1*, *Acot2*, *Cyp4a31*, *Cyp17a1*, *Fgf21*, *Inhbb*, *Pdk4*, *Pparγ* and *Vnn1*) were expressed differently between the DHEA and control groups. To verify the expression of these 9 genes in the liver of female and male offspring, the sample size was expanded to six mice per group. As assessed by mRNA level, the expression of each of *Pparγ*, *Fgf21*, *Pdk4*, *Inhbb*, *Acot1* and *Acot2* was significantly upregulated in DHEA female offspring, whereas *Cyp17a1* was significantly downregulated (Fig. 5A). Unlike in female offspring, the expression of these genes in male offspring did not differ significantly between the DHEA and control groups (Fig. 5B).

## Discussion

DHEA is a metabolic intermediate in the biosynthesis of androgen and is one of the many drugs, including testosterone and letrozole, that are used for animal models of PCOS by prenatal or prepubertal exposure (Luchetti *et al.* 2004, Manneras *et al.* 2007). In our study, PCOS was induced by the administration of DHEA (6 mg/100 g body weight) for 20 consecutive days in prepubertal BALB/c mice (Sander *et al.* 2006, Abramovich *et al.* 2012, Zhang *et al.* 2013). This dose of DHEA ensures a hyperandrogenized status equivalent to that found in women with PCOS (Lee *et al.* 1991, Luchetti *et al.* 2004, Solano *et al.* 2006). An increase in fat and stromal tissue is observed in ovaries of prepubertal mice from DHEA-treated mice (Luchetti *et al.* 2004). Furthermore, the ovarian cortex of treated mice has more cysts, which show a thin layer of theca cells and compacted granulosa cells with the absence of a vascularized theca interna. Decreased insulin sensitivity, increased ovarian oxidative stress, altered serum estradiol and progesterone levels, and ovarian immunosuppressor prostaglandin E were also apparent in prepubertal hyperandrogenism after DHEA treatment (Luchetti *et al.* 2004, Elia *et al.* 2006). Characteristics of DHEA-treated mice, especially hyperandrogenism, are similar to those exhibited by women with PCOS. Therefore, we chose this PCOS mouse model to assess the growth and metabolic characteristics of PCOS offspring.

We examined the stage of cyclicity, ovarian morphology, hormone levels and changes in metabolism and fertility in mice after 20 consecutive days of DHEA injection. The results showed that all mice from the DHEA group, whose ovaries were markedly swollen and had multiple follicular cysts, were completely acyclic and remained in constant estrous. Levels of testosterone, TG and CHO were significantly increased, and the weight gain and fat mass were significantly greater than those in the control group, as noted in our previous study (Huang *et al.* 2015).

A number of animal studies have shown that excessive intrauterine androgen exposure results in many problems in offspring, such as excessive weight gain, visceral and subcutaneous fat accumulation, increased fat cell volume, impaired glucose tolerance, decreased insulin sensitivity and lipid metabolism disorders (Demissie *et al.* 2008, Roland *et al.* 2010, Yan *et al.* 2013). PCOS mothers have a significantly higher prevalence of birthing both small- and large-for-gestational-age newborns, and birth length for the latter is significantly greater than that of controls (Sir-Petermann *et al.* 2005). Assessment of prepubertal daughters of patients with PCOS revealed significantly greater bone age and basal height compared with prepubertal girls in the control group, although body mass index did not differ significantly between the groups (Battaglia *et al.* 2002). However, the study included only 15 prepubertal offspring of patients with PCOS and 10 control prepubertal girls, and longer-term follow-up studies concerning offspring of PCOS women are rare. A rat model in which PCOS was induced by prenatal exposure to testosterone revealed no significant differences in body weight of offspring at birth and postnatal day 15, but when compared with controls at 30, 45 and 60 days of age and in adulthood, the body weight of PCOS rat offspring was significantly greater (Noroozzadeh *et al.* 2015). However, none of the studies mentioned previously distinguished the female and male offspring. We found that, although the body weight of female offspring of DHEA mice was significantly greater than that of the control group from postnatal week 1 into adulthood, the body weight of the male offspring of the DHEA group in the period of 1–4 weeks after birth was also significantly greater; however, there was no significant difference from weeks 5–12. The reason for this might be that, before puberty, the body weight of offspring was influenced by PCOS mothers as a consequence of parenting factors related to the physical and pathological state of this syndrome. During adolescence and adulthood, however, female body weight is more easily affected by ovarian hormones. Ovarian physiopathological characteristics of female offspring might be inherited from the PCOS mother, and resulting in greater body weight gain. On the other hand, because there was an inverse relation between the average body weight and litter size (Reddy & Reddy, 1982), the significantly smaller litter size of PCOS dams may have the consequence of increased mean body weight of offspring simply due to increased access to maternal resources, both in utero and postnatally. It is hard for us to completely exclude the maternal resource factor. This could be a confounding factor, and it is a limitation of our study.

In this study, mild glucolipid metabolism disorders existed in the female offspring in the DHEA group, such as increased body fat and serum TG and impaired glucose tolerance. Decreased energy consumption might be one of the causative factors. The phenomenon may also be due to epigenetic and pathophysiological changes in metabolic tissue resulting from the female fetus’s exposure to elevated testosterone level (Abbott *et al.* 2010). Studies with rhesus monkeys showed that androgen treatment in different gestational periods yields different outcomes. Excess androgen in early gestation programs both hyperinsulinemia from adiposity-dependent insulin resistance and preferential accumulation of visceral adiposity, whereas androgen excess later in gestation decreases insulin sensitivity and increases non-visceral abdominal fat, although insulin secretion is unaffected (Bruns *et al.* 2007, Abbott *et al.* 2010). Prenatal androgenization of female rhesus monkeys may modify DNA methylation patterns in both infant and adult visceral adipose tissues (Xu *et al.* 2011), suggesting that changes in offspring metabolism may be related to alterations in DNA methylation or other epigenetic modifications.

Because of the increased catabolism of fat associated with insulin resistance, however, the consequent excessive free fatty acids in the circulation are transported to the liver. When oxygenolysis in liver mitochondria is insufficient to catabolize the excess free fatty acids, the remaining free fatty acids accumulate in the liver, resulting in an increase in TG level (Tolman *et al.* 2004). Pyruvate dehydrogenase kinase (PDK) 4 is a member of enzymes that inhibit the activity of the pyruvate dehydrogenase complex, and PDK links the glycolytic degradation of glucose to the tricarboxylic acid cycle through the rate-limiting and physiologically irreversible oxidative decarboxylation of pyruvate (Sugden & Holness 2006). PDK4 is primarily expressed in liver, heart and skeletal muscle. A high-fat diet significantly increases the level of PDK4 in skeletal muscle and cardiac muscle of mice. In addition, diabetes, hunger or fatty acids converted by sugars as energy source also upregulate PDK4 expression significantly (Feige & Auwerx 2007, Connaughton *et al.* 2010, Rinnankoski-Tuikka *et al.* 2012, Zhang *et al.* 2012). In our study, the increased expression of *Pdk4* in the DHEA group inhibited the activity of pyruvate dehydrogenase, which may be one of the reasons for the observed decrease in insulin sensitivity.

Peroxisome proliferator-activated receptor γ (PPARγ), a member of the nuclear hormone receptor superfamily, is a class of ligand-activated transcription factors that play a key role in glucose homeostasis and maintaining lipid metabolism. It was traditionally believed that one subtype of PPARγ is expressed in adipose tissue, in which it regulates preadipocyte differentiation and promotes adipocyte maturation (He *et al.* 2003). However, some studies have shown that PPARγ also plays a functional role in liver, muscle and other tissues (Vidal-Puig *et al.* 1996, Norris *et al.* 2003). Activated PPARγ increases the sensitivity of peripheral tissues to insulin, decreases insulin resistance and lowers blood glucose (Lazar 2005, Badman *et al.* 2007, King *et al.* 2007) through signaling pathways of acyl-CoA thioesterases and fibroblast growth factor 21 (FGF21) (Lazar 2005, Badman *et al.* 2007, King *et al.* 2007). These thioesterases comprise a class of enzymes that catalyze the conversion acyl-coenzyme A to free fatty acids via hydrolysis, which is regulated by PPARγ and play an important role in fatty acid synthesis and degradation (Hunt & Alexson 2002). FGF21, which was recently discovered, is a potent regulator of glucose uptake in adipocytes, regulating both glucose and lipid metabolism through fatty acid oxidation in the liver (Kharitonenkov *et al.* 2005). Overexpression of FGF21 in transgenic mice prohibits diet-induced obesity. FGF21 treatment can reduce body weight, blood glucose and blood lipids and reverse hepatic steatosis in diabetic mice (Kharitonenkov *et al.* 2005, Xu *et al.* 2009). The expression of *Pparγ*, *Acot1*, *Acot2* and *Fgf21* were increased in the liver of PCOS offspring mice, which may be related to compensatory adjustment for confrontation of dyslipidemia or protein resistance of the above-mentioned molecules. Similarly, one study showed that, when the body contained higher fatty acids, as a result of diabetes or a high-fat diet, the expression of *Acot1* and *Acot2* was upregulated in liver, thereby promoting the catabolism of fatty acids (Yamada *et al.* 2003). Human serum FGF21 levels also correlate positively with body mass index, as evidenced by the significantly increased level of serum FGF21 in obese patients (Zhang *et al.* 2008, Dushay *et al.* 2010). Overall, *Pparγ*, *Acots*, *Fgf21* and *Pdk4* all play important roles in regulating glucose and lipid metabolism, and the regulatory mechanisms are complex because the expression of these four genes is affected by many factors such as hunger and diet. Changes in the expression of these genes may be closely associated with the observed glycolipid metabolism disorder in offspring of DHEA-induced mice, and the increased levels of PPARγ and FGF21 in the liver of PCOS offspring may be a compensatory mechanism that maintains normal glucose and lipid metabolism.

The gene, *CYP17A1*, encodes an enzyme of the cytochrome P450 superfamily, and it plays a role in cortisol production in humans and animals (Lima *et al.* 2015). In humans, *CYP17A1* is responsible for the synthesis of P450c17, which is a key enzyme in the steroidogenic pathway for the metabolic conversion of progesterones to adrenal androgens and their subsequent conversion to testosterone (Fan *et al.* 2009, Kosaka *et al.* 2014). Androgens serve as precursors to estrogens, so normal estrogen signaling also depends on *CYP17A1*, which plays a very important role in many other physiological and pathological processes such as vascular endothelium repair and lipid metabolism. Moreover, CYP17A1 is also involved in glucose metabolism and insulin-related signal transduction pathways (Dai *et al.* 2015). Studies have shown increased expression of *CYP17* genes in theca cells derived from PCOS women and an endogenous hyperandrogenism rat model of PCOS (Wood *et al.* 2003, Li *et al.* 2013). However, lipopolysaccharide levels in ovarian follicular fluid of dairy cows influence *CYP17* expression in theca cells (Magata *et al.* 2014). In follicles with a high level of lipopolysaccharides, *CYP17* expression was lower (Magata *et al.* 2014). This study showed that *Cyp17a1* mRNA level was significantly decreased in liver tissue of DHEA female offspring, which may be related to body weight gain, increased body fat and TG content or to impaired glucose tolerance. In conclusion, we found that the average litter size was significantly smaller in the DHEA group, and the female offspring in the DHEA group showed sustained greater body weight, increased body fat and TG content in serum and liver; decreased energy expenditure and oxygen consumption; and impaired glucose tolerance. In liver tissue of female offspring of the DHEA group, the glucolipid metabolism–related genes such as *Pparγ*, *Acot1/2*, *Fgf21*, *Pdk4* and *Inhbb* were upregulated, whereas *Cyp17a1* was significantly downregulated.

## Supplementary data

This is linked to the online version of the paper at http://dx.doi.org/10.1530/REP-16-0081.

## Declaration of interest

The authors declare that there is no conflict of interest that could be perceived as prejudicing the impartiality of the research reported.

## Funding

This work was supported in part by grants from the Ministry of Science and Technology of China (973 program; 2014CB943203), ‘Reproductive health and major birth defects prevention and control research’ Key Special Fund (2016YFC1000601), the National Natural Science Foundation General Program (81300543, 81571400 and 31501201), the Beijing Nova Program (xxjh2015011), the Specialized Research Fund for the Doctoral Program of Higher Education (20120001130008) and special projects of the 2015 Science and Technology Innovation Base Cultivation and Development (Z151100001615023).

## Author contribution statement

The principal investigators Y Zhao, R Li, Y Yu and J Qiao take primary responsibility for the paper and contributed to the conception, design and coordination of the research. Y Huang and J-M Gao performed the experiment and data analysis. C-M Zhang and H-C Zhao contributed to the acquisition of reagents/materials/analysis tools. Y Huang and J-M Gao contributed to writing the manuscript. Y Zhao, R Li and Y Yu contributed to the revision of the article, and the final version was approved by all the authors.
